# Editorial: Women in science: headache

**DOI:** 10.3389/fpain.2023.1324072

**Published:** 2023-11-09

**Authors:** Danielle D. DeSouza, Megan Bennett Irby, Catherine S. Hubbard

**Affiliations:** ^1^ Acacia Research Center, Acacia Clinics, Sunnyvale, CA, United States; ^2^ Neurology & Neurological Sciences, Stanford University, Stanford, CA, United States; ^3^ Department of Health and Exercise Science, Wake Forest University, Winston-Salem, NC, United States; ^4^ Department of Neuroscience, Medical University of South Carolina, Charleston, SC, United States

**Keywords:** migraine, headache, women researchers, chronic headache, chronic migraine, episodic migraine, women

**Editorial on the Research Topic** Women in science: headache

Science knows no gender. Yet, the representation of women in the global scientific community tells a different story. Despite the pressing need for diverse perspectives, UNESCO reports that women comprise fewer than 30% of researchers globally. To reshape traditional mindsets, it is imperative that we challenge stereotypes and promote gender equality by ensuring that women are equally represented and celebrated in research. In the rapidly advancing and ever-evolving realm of scientific discovery, ensuring gender equality is not merely a matter of principle—it's a fundamental driver of progress and an essential catalyst for fueling transformative change and pioneering innovations. In this Research Topic, we honor the invaluable contributions of women trailblazers in headache research. The selected papers all share one criterion: a woman stands as the lead or senior author, underscoring their pivotal role in advancing the field. Collectively, their research provides crucial insights into the multifaceted domain of headache, offering groundbreaking findings that advance our basic understanding and treatment of these conditions.

As part of this Research Topic, an observational study by Deodato et al. investigated pain hypersensitivity in individuals with chronic migraine. The research examined the effects of three months of treatment involving OnabotulinumtoxinA therapy, physical therapy (PT), or a combination of both therapies. The study meticulously examined pressure pain thresholds (PPT) in various muscles within the trigeminocervical area while also monitoring headache parameters, including attack frequency, duration, and pain intensity. The results yielded valuable insights. While all three groups demonstrated significant decreases in headache frequency and attack duration, only the combined treatment of OnabotulinumtoxinA and PT demonstrated significant improvements in pain intensity and PPT compared to single-therapy conditions. The authors suggested that pain modulation can be optimized in patients with chronic migraine when therapies are combined, with clinical effects impacting headache features and pressure hyperalgesia.

In another publication, Mungoven et al. assessed alterations in brain structure in individuals with episodic migraine. The authors previously demonstrated altered trigeminal nerve anatomy in episodic migraine sufferers; however, it was unclear if these alterations were also associated with changes in the brainstem or higher brainstem and cortical regions. Using a specialized structural magnetic resonance imaging (MRI) approach called diffusion tensor imaging (DTI), it was found that two regional measures of structure, fractional anisotropy (FA) and mean diffusivity (MD), were inversely related in the regions examined such that lower FA in the trigeminal nerve was significantly related to higher MD in the brainstem and multiple cortical brain regions. Importantly, this relationship was observed in both individuals with episodic migraine and controls but was disrupted in the migraine group in the brainstem periaqueductal gray (PAG) and primary visual (V1) cortices. As these two brain regions are commonly implicated in migraine pathophysiology, the authors suggested that alterations in their anatomy may contribute to altered pain processing in migraine.

This Research Topic also includes an Opinion Article by Smirnoff, addressing the safety of using OnabotulinumtoxinA for managing chronic migraine during pregnancy. In her article, Smirnoff explores the known risks, safety considerations, and outcomes associated with this treatment option. While the FDA currently assigns a pregnancy rating of C for OnabotulinumtoxinA due to a lack of comprehensive studies in pregnant women, Smirnoff highlights findings from both animal and human studies suggesting it remains a very strong option for the management of chronic migraine in pregnancy. This article also underscores the need for further research to fully establish OnabotulinumtoxinA safety and efficacy for chronic migraine during pregnancy.

To conclude this Research Topic, Dumkrieger et al. make a significant contribution to the ongoing efforts to create diagnostic tools based on machine learning classifiers to differentiate various headache subtypes. They do so by adopting a multidimensional approach, integrating MRI assessments of brain structure and function (functional connectivity), headache questionnaires, and cognitive tests. Their primary objective was to distinguish individuals with persistent post-traumatic headache (PPTH) from those experiencing migraine, both of which exhibit similar clinical characteristics. Remarkably, the team achieved an 87.8% accuracy in distinguishing between the two headache types, a notable improvement compared to previously published models that attained 78% accuracy but did not incorporate functional connectivity data. They further validate the advantage of including functional connectivity measures in their model using other conservative modeling approaches. This study underscores the promise of advanced imaging methods and machine learning in distinguishing between different types of headache disorders.

The four articles showcased in this Research Topic highlight the transformative work of women researchers in the field of headache. Their collective efforts and innovative approaches have advanced our understanding of topics ranging from pain modulation and the neural underpinnings of migraine to the safety of treatments during pregnancy and the nuanced differentiation of headache subtypes. As we celebrate their accomplishments, it is essential to recognize that gender equality, diverse perspectives, and the relentless pursuit of equity in the scientific realm are vital for the continued progress of science. By supporting and promoting the work of women researchers in the field, we move ever closer to a future where scientific achievements are truly inclusive and equitable. In reflecting on their pioneering research, we are reminded of Marie Curie's words: “I was taught that the way of progress was neither swift nor easy” (1). Their perseverance and unwavering determination echo this sentiment, as does the collective effort of countless women researchers working tirelessly every day to forge a future where scientific pursuits transcend gender boundaries.
